# The mycobacterial antibiotic resistance determinant WhiB7 acts as a transcriptional activator by binding the primary sigma factor SigA (RpoV)

**DOI:** 10.1093/nar/gkt751

**Published:** 2013-08-28

**Authors:** Ján Burian, Grace Yim, Michael Hsing, Peter Axerio-Cilies, Artem Cherkasov, George B. Spiegelman, Charles J. Thompson

**Affiliations:** ^1^Department of Microbiology and Immunology, University of British Columbia, Vancouver, Canada V6T 1Z3, ^2^Centre for Tuberculosis Research, University of British Columbia, Vancouver, Canada V6T 1Z3 and ^3^Vancouver Prostate Centre, University of British Columbia, Vancouver, Canada V6T 1Z3

## Abstract

Tuberculosis therapeutic options are limited by the high intrinsic antibiotic resistance of *Mycobacterium tuberculosis.* The putative transcriptional regulator WhiB7 is crucial for the activation of systems that provide resistance to diverse antibiotic classes. Here, we used *in vitro* run-off, two-hybrid assays, as well as mutagenic, complementation and protein pull-down experiments, to characterize WhiB7 as an auto-regulatory, redox-sensitive transcriptional activator in *Mycobacterium smegmatis*. We provide the first direct biochemical proof that a WhiB protein promotes transcription and also demonstrate that this activity is sensitive to oxidation (diamide). Its partner protein for transcriptional activation was identified as SigA, the primary sigma factor subunit of RNA polymerase. Residues required for the interaction mapped to region 4 of SigA (including R515H) or adjacent domains of WhiB7 (including E63D). WhiB7’s ability to provide a specific spectrum of antibiotic-resistance was dependent on these residues as well as its C-terminal AT-hook module that binds to an AT-rich motif immediately upstream of the −35 hexamer recognized by SigA. These experimentally established constrains, combined with protein structure predictions, were used to generate a working model of the WhiB7–SigA-promoter complex. Inhibitors preventing WhiB7 interactions could allow the use of previously ineffective antibiotics for treatment of mycobacterial diseases.

## INTRODUCTION

*Mycobacterium tuberculosis *(*Mtb*), the causative agent of tuberculosis (TB), is the leading cause of death due to bacterial infection worldwide. One of the major obstacles to controlling the TB pandemic is that *Mtb *is intrinsically resistant to the majority of clinically available antibiotics (1). We focus on the putative transcriptional regulator WhiB7, a critical component for the activation of intrinsic antibiotic resistance systems in *Mtb, Mycobacterium bovis *BCG, *Mycobacterium smegmatis* and *Streptomyces lividans *(2) as well as *Rhodococcus jostii *(Ramon-Garcia *et al.*, unpublished).

The *whiB *gene was first identified in studies of *Streptomyces coelicolor *mutants whose white aerial mycelium was unable to divide and differentiate into grey spores (3). WhiB orthologs are found across actinomycetes and proposed to serve as transcriptional regulators with a role in cell division (4,5). Many homologous *whiB*-like (*wbl*) genes, found exclusively within the Actinomycete taxon, form the WhiB family of putative transcriptional regulators (6,7). In *Mtb*, seven *wbl* genes have essential roles for growth (*whiB1* and *whiB2*) (8,9) or play critical roles in redox balance (*whiB3, whiB4* and *whiB7*), antibiotic resistance (*whiB7*), virulence (*whiB3, whiB4, whiB5* and *whiB6*), dormancy (*whiB1*) or reactivation (*whiB1* and *whiB5*) (2,8–16). Transcription of *whiB *genes is induced by a variety of physiological and stress conditions (2,17–19). As their mutant phenotypes indicate important roles in *Mtb *physiology, adaptation, and cell division, the biochemical functions of WhiB proteins are under active investigation.

**Figure 1.**
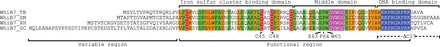
Conservation of WhiB7 sequence and regions. Sequence comparison of WhiB7 proteins from *M. tuberculosis *(TB), *M. smegmatis* (SM), *R. jostii *(RH) and *S. coelicolor *(SC). Predicted conserved structural features are highlighted; four conserved cysteines in red, the WhiB specific tryptophan-containing turn in pink, and the AT-hook motif in blue. Other fully conserved residues are in green and chemically similar residues are in orange. Residues either mutated or deleted in this study are indicated and numbered according to the *M. smegmatis* sequence.

Wbl proteins all share three sequence motifs including an N-terminal domain with four conserved cysteines, a unique G(V/I)WGG turn and a C-terminal block of basic amino acids. The cysteines coordinate an iron-sulphur (FeS) cluster (20) (holo protein) or may form intramolecular disulphide bonds (apo protein), whereas the tryptophan region and the C-terminal basic amino acids was predicted to form a helix turn helix like structure that may allow DNA binding (4,21). In the case of WhiB7, the C-terminus contains a distinct AT-hook, an amino acid motif that binds the minor groove of AT-rich DNA (Figure 1) (2,22). Apo forms of WhiB1, WhiB2, WhiB3 and WhiB4 bind DNA more tightly than their respective holo proteins, with oxidized apo proteins having higher affinity for specific recognition sequences than their reduced forms (9,12,15,21). DNase footprinting has demonstrated sequence-specific DNA binding for apo-WhiB1, and *in vitro *transcription demonstrated that it acts as a repressor (9,23). However, WhiB2 (8), WhiB3 (15,24), WhiB5 (11) and WhiB7 (2,10) are hypothesized to be transcriptional activators based on microarrays, mutant phenotypes and other indirect evidence. A second thioredoxin-like function is suggested by observations that all *Mtb *WhiBs lacking an FeS cluster (with the exception of WhiB2) are able to reduce insulin disulphide in the presence of dithiothreitol (DTT) (20). Finally, a third, chaperone-like, function has been inferred from observations that WhiB2 prevents aggregation of several model protein substrates (25). Surprisingly, WhiB2’s cysteines are dispensable for its chaperone activity but are required to complement the *whiB2* mutation (26). More analysis is required to understand the complex molecular functions of this interesting family of proteins.

WhiB7, the focus of our study, is required for resistance to a diverse array of antibiotics with different structures and targets. The *whiB7* sensitivity spectrum includes macrolides, tetracyclines, lincosamides, pleuromutilins, phenicols and some aminoglycosides (2,10). Microarray studies suggest that the known antibiotic resistance genes *erm*, *tap* and *eis* are part of the WhiB7 regulon (2). The fact that activation of *whiB7* transcription does not generate resistance to all antibiotics that induce its expression suggests that it may have other functions (10). *whiB7* is upregulated not only by antibiotic treatment but also by certain physiological stresses including iron starvation, heat shock and entry intro stationary phase (18). Importantly, *whiB7* is globally upregulated after *Mtb* infects macrophages in culture (either resting or activated) (27), as well as in the lungs of infected mice (19), suggesting a role in virulence. Interestingly, three genes within the WhiB7 regulon, *eis*, *erm* and *tap *not only contribute to antibiotic resistance (28–30) but also modulate the host immune response (31–33). Temporal analysis of the *Mtb *transcriptome during macrophage infection shows that *whiB7* is one of the first genes expressed after entry into this new and hostile environment (34). The interplay between physiological stress, antibiotic resistance and *whiB7* has recently been reviewed (35).

Microarray and promoter-reporter analyses led to the hypothesis that WhiB7 is an autoregulatory transcriptional activator (2,10). The *whiB7* promoter contains a conserved AT-rich sequence motif required for optimal induction, implying that WhiB7 targets its own promoter via its C-terminal AT-hook motif (10). Here, we show that WhiB7 increases transcription from its promoter *in vitro* in a redox-sensitive manner, the first direct biochemical evidence that a WhiB protein promotes transcription. We also use two-hybrid, co-expression, mutagenesis and complementation assays to investigate the mechanism of WhiB7-mediated transcriptional activation.

## MATERIALS AND METHODS

### Bacterial strains and growth conditions

Unless otherwise specified *Escherichia coli *strains were grown in LB (Sigma) at 37°C, shaking at 200 rpm. *M. smegmatis *mc^2^155 and its derivatives were grown in Middlebrook 7H9 (BD) supplemented with 10% (v/v) ADC (BD), 0.2% (v/v) glycerol and 0.05% (v/v) tyloxapol (Sigma) at 37°C either shaking in flasks at 200 rpm or rolling in test tubes. If needed, appropriate antibiotics were added to the media; hygromycin 50 µg/ml, kanamycin 30 µg/ml, chloramphenicol 34 µg/ml, tetracycline 12.5 µg/ml and ampicillin 200 µg/ml.

### Cloning

Cloning of bait and target constructs was done in *E. coli *MRF’ Kan as specified by Stratagene. *E. coli *TOP10 was used for all other cloning. Unless otherwise specified, PCR was performed using Easy-A Hi-Fi polymerase (Agilent) according to manufacturer instructions. Reactions included 3% (v/v) dimethyl sulphoxide. Restriction enzymes were from New England Biolabs; digests were performed according to manufacturer instructions. Ligations were performed using T4 DNA ligase (Invitrogen) at room temperature for 30 min. Sequencing of vector constructs was performed at the UBC Vancouver campus NAPS unit (http://naps.msl.ubc.ca/). When necessary, custom primers were used to sequence; they are indicated by a ‘seq’ tag in the primer list. All oligonucleotides used are listed in Supplementary Table S1. All constructed vectors used are described in Table 1, with details of their construction provided in the Supplementary Materials and Methods.

**Table 1.** Strains and plasmids used

Strains	Description	Reference
*E. coli*		
TOP10	Cloning and plasmid maintenance	Invitrogen
MRF’ Kan	Cloning and plasmid maintenance for pBT, pTRG and their derivatives; Kan^R^	Stratagene
Rosetta2 (DE3)	Protein expression strain containing rare tRNAs; Cm^R^	Novagen
BacterioMatch II reporter	Reporter strain for the BacterioMatch II two-hybrid system; Kan^R^	Stratagene
*M. smegmatis* mc^2^155		
parental	Unmodified laboratory strain	(36)
* whiB7* KO	*whiB7* (genomic region 2031710–2032094) replaced by hygromycin resistance; Hyg^R^	(10)
FB7	N-terminal 3xFLAG tag and *whiB7* fusion	This study
Sig515	*sigA *mutant expressing SigA R515H; Hyg^R^	This study
Plasmids		
pMS497GFP	pMycVec1 derivative with the *whiB7* promoter driving *gfp *expression. Used as the PCR template for *whiB7* promoter; Kan^R^	(10)
pMS483GFP	pMycVec1 derivative with the *whiB7* promoter, lacking the AT-rich region, driving *gfp *expression. Used as the PCR template for a truncated promoter *whiB7* promoter lacking the putative WhiB7 binding site; Kan^R^	(10)
pMS689GFP	pMycVec1 derivative with the *whiB7* promoter driving *gfp *expression. Used as the PCR template for alternate *whiB7* promoter templates; Kan^R^	(10)
pETB7sm	pET19b derivative for the expression of 10xHis-WhiB7; Amp^R^	This study
pETB7epy	Mutant of pETB7sm expressing 10xHis-WhiB7 W65Y; Amp^R^	This study
pETB7vey	Mutant of pETB7sm expressing 10xHis-WhiB7 E63V P64E W65Y; Amp^R^	This study
pETB7d	Mutant of pETB7sm expressing 10xHis-WhiB7 E63D; Amp^R^	This study
pETB748	Mutant of pETB7sm expressing 10xHis-WhiB7 C48A; Amp^R^	This study
pETB74548	Mutant of pETB7sm expressing 10xHis-WhiB7 C45A C48A; Amp^R^	This study
pET19b	T7 RNAP promoter driven protein expression vector; Amp^R^	Novagen
pSigA	pColDuet-1 derivative for the expression of strepII-SigA_C170_ of SigA; Kan^R^	This study
pSigAB7	pColDuet-1 derivative for the co-expression of 10xHis-WhiB7 and strepII-SigA_C170_; Kan^R^	This study
pSigAB748	Mutant of pCDR43B7 co-expressing strepII-SigA_C170_ and 10xHis-WhiB7 C48A; Kan^R^	This study
pSigAB74548	Mutant of pCDR43B7 co-expressing strepII-SigA_C170_ and 10xHis-WhiB7 C45A C48A; Kan^R^	This study
pR4B7	pColDuet-1 derivative for the co-expression of 10xHis-WhiB7 and strepII-SigA_C82_; Kan^R^	This study
pColaDuet-1	T7 RNAP promoter driven protein co-expression vector; Kan^R^	Novagen
pBTW7	Bait WhiB7 fused to the C-terminus of λcI; Cm^R^	This study
pBTW7ΔC19	Bait WhiB7 lacking the AT-hook fused to the C-terminus of λcI; Cm^R^	This study
pBTW7mid	Bait WhiB7 fragment, amino acids 50-80, fused to the C-terminus of λcI; Cm^R^	This study
pBTW7epy	Mutant of pBTW7ΔC19 expressing an AT-hookless WhiB7 W65Y fused to the C-terminus of λcI; Cm^R^	This study
pBTW7vey	Mutant of pBTW7ΔC19 expressing AT-hookless WhiB7 E63V P64E W65Y fused to the C-terminus of λcI; Cm^R^	This study
pBTW7d	Mutant of pBTW7ΔC19 expressing AT-hookless WhiB7 E63D fused to the C-terminus of λcI; Cm^R^	This study
pBTW71d	Mutant of pBTW7ΔC19 expressing AT-hookless WhiB7 E71D fused to the C-terminus of λcI; Cm^R^	This study
pBTW748	Mutant of pBTW7ΔC19 expressing AT-hookless WhiB7 C48A fused to the C-terminus of λcI; Cm^R^	This study
pBTW74548	Mutant of pBTW7ΔC19 expressing AT-hookless WhiB7 C45A C48A fused to the C-terminus of λcI; Cm^R^	This study
pLGF2	Positive control bait vector with LGF2 fused to the C-terminus of λcI; Cm^R^	Stratagene
pBT	Empty bait vector expressing λcI; Cm^R^	Stratagene
pSigASM	Target SigA fused to the C-terminus of the α-subunit of RNAP; Tet^R^	This study
pTRG170	Target region 4.2 of SigA fused to the C-terminus of the α-subunit of RNAP; Tet^R^	This study
pTRG170.515	Target region 4.2 of SigA with the R515H mutation fused to the C-terminus of the α-subunit of RNAP; Tet^R^	This study
pGAL11	Positive control target vector expressing Gal11 fused to the C-terminus of the α-subunit of RNAP; Tet^R^	Stratagene
pTRG	Target vector expressing the α-subunit of RNAP; Tet^R^	Stratagene
pFB7	pMV261 derivative for constitutive expression of *whiB7* with a N-terminal 3xFLAG epitope; Kan^R^	This study
pFB7AT	pMV261 derivative for constitutive expression of w*hiB7* with a N-terminal 3xFLAG epitope and lacking the C-terminal AT-hook; Kan^R^	This study
pFB7d	pMV261 derivative for constitutive expression of *whiB7* with a N-terminal 3xFLAG epitope and a WhiB7 E63D mutation; Kan^R^	This study
pB7fun	pMV261 derivative for constitutive expression of the WhiB7 functional region (WhiB7ΔN19C6); Kan^R^	This study
pMV261	A mycobacterial multi-copy vector containing the constitutively active HSP60 promoter upstream of a multiple cloning site; Kan^R^	(37)

Hyg^R^, hygromycin resistance; Kan^R^, hygromycin resistance; Amp^R^, ampicillin resistance; Cm^R^, chloramphenicol resistance; Tet^R^, tetracycline resistance.

### Purification of RNA polymerase

*M. smegmatis *was grown in 7H9 to an optical density at 600 nm of about 1. Cells were pelleted by centrifugation at 4000 *g* for 30 min. This process was repeated enough times to gain a combined pellet weighing >30 g. RNA polymerase (RNAP) was isolated as previously described (38) with the exceptions that the cells were sonicated 18 times and active fractions from the DNA-cellulose column were not concentrated. Active fractions were adjusted to a final glycerol concentration of 50% (v/v) and then stored at −80°C for direct use.

### Purification of WhiB7

*E. coli* Rosetta2 (DE3) cells were used to express WhiB7 from pETB7sm. Culture at an OD 600 nm of about 0.4 were induced by the addition of 0.3 mM isopropyl-beta-D-thiogalactopyranoside (IPTG) and incubated for 17 h at 16°C. Cells were harvested by centrifugation and lysed in lysis buffer [50 mM Na_2_PO_4_, 300 mM NaCl, 10 mM imidazole, 50 µg/ml phenylmethanesulphonylfluoride, 5 mM 2-mercaptoethanol (pH 8)] by sonication. Lysate supernatant was isolated by ultra-centrifugation and filtered through a 0.45 µm filter. Supernatant was loaded onto Ni-NTA resin (Qiagen) held at 4°C for His-tagged protein isolation. The column was then washed with six 10 column volumes of wash buffer [50 mM Na_2_PO_4_, 300 mM NaCl (pH 8)] containing an increasing amount of imidazole (50, 60, 70, 80, 90 and 100 mM). Finally elution buffer (wash buffer + 250 mM imidazole) was applied to the column. This resulted in a clear, brown eluate. Fractions that were visibly dark brown were pooled, DTT was added to a final concentration of 2 mM, and aliquots were immediately frozen in liquid nitrogen and stored at −80°C until use. A more detailed protocol is provided in the Supplementary Materials and Methods.

### *In vitro* run-off assay

DNA templates were pre-incubated for 2 min at 37°C in transcription buffer [10 mM HEPES (pH 8), 10 mM magnesium acetate, 80 mM potassium acetate, 0.1 mM DTT, 0.1 mg/ml acetylated BSA]. WhiB7 (various concentrations, as indicated) or elution buffer were added to the DNA solution and incubated an additional 2 min at 37°C. In experiments designed to test the effect of oxidation on WhiB7 activity, the protein was pre-incubated in the presence or absence of 7 mM diamide before adding it to the run-off reaction mix. Owing to the staggered running of the transcription reactions, the length of diamide treatment ranged from 15 to 26 min for the lowest to highest concentration of WhiB7. Transcription was initiated with RNAP and after 2 min at 37°C, a mixture of NTP and heparin was added to arrest additional transcriptional initiation. All reactions had a final volume of 10 μl and contained: transcription buffer, 16 nM template, 80 nM RNAP, 50 μg heparin ml^−^^1^, 400 μM CTP, 400 μM UTP, 400 μM ATP, 5 μM GTP and 111 kBq [α-^32^P] GTP. Transcripts were elongated for 5 min at 37°C and then terminated by the addition of 5 μl of loading buffer [1.5X transcription buffer with 0.1% (w/v) bromophenol blue, 0.1% (w/v) xylene cyanol and 7 M urea]. Transcripts were electrophoresed through 8% denaturing acrylamide gels and imaged using a Storage Phospor screen (Amersham Biosciences), scanned by a Typhoon 9400 (Amersham Biosciences) and quantified using ImageQuant 5.2 software (Amersham Biosciences). Heparin was from Sigma, NTPs were obtained from Amersham Biosciences and [α-^32^P]GTP (111 TBq mmol^−1^) was from PerkinElmer Life Sciences.

### Two-hybrid assay

The bait and target vectors were co-transformed into the BacterioMatch II two-hybrid reporter strain. Cells recovered for 1 h at 37°C in 1 ml of LB broth. Cells were then pelleted by centrifugation and washed twice with 1 ml of M9^+^ His-dropout broth [866 ml of Salt Base (55 mM Na_2_HPO_4_, 25.5 mM KH_2_PO_4_, 9.9 mM NaCl, 21.6 mM NH_4_Cl), 130 ml of Solution I (3.08% glucose, 1.54 mM adenine, 5.92 g/l -His SO supplement (Clontech #630415) and 4 ml of Solution II (0.25 M MgSO_4_, 0.25 M Thiamine, 2.5 mM ZnSO_4_, 25 mM CaCl_2_)]. Transformants were incubated an additional hour in 1 ml of M9^+^ His-dropout broth, plated on M9^+^ His-dropout agar, and grown overnight at 37°C. Individual co-transformants were inoculated into in 3 ml of Nonselective Screening Medium (NSM) [M9^+^ His-dropout broth supplemented to 50 µM IPTG] and grown overnight. Cultures were diluted as indicated and 8 µl was spotted on both NSM and selective screening medium (SSM) (5 mM 3-amino-1,2,4-triazole in NSM) agar plates and grown for 24 h at 37°C. Plates were then incubated for an additional 24 h at room temperature and pictures taken.

### WhiB7 and SigA co-expression and pull-down

pSigAB7 was transformed into *E. coli* Rosetta2 (DE3). The expression and Ni-NTA purification were carried out as described for the purification of WhiB7 with the exception that the Ni-NTA was washed with 6 column volumes of wash buffer containing 50 mM imidazole. The Ni-NTA eluate was subsequently applied to Strep-Tactin sepharose resin (IBA) and purified according to manufacturer’s instructions. Samples were taken throughout the purification process and were mixed 1:1 with 4× Sample buffer [0.25 M Tris–HCl, 0.28 M sodium dodecyl sulphate, 40% (v/v) glycerol, 20% (v/v) 2-mercaptoethanol (pH 6.8)] and heated at 100°C for 25 min. The samples (7.5 µl) were then separated using a 10% Tricine-SDS–PAGE gel as described by Schagger (39). Proteins were visualized using GelCode (Thermo Scientific) according to manufacturer’s instruction. The Micro BCA protein assay kit (Thermo Scientific) was used according to manufacturer instructions to quantify the resulting WhiB7–SigA complex at 0.767 ± 0.021 µg/ml.

pSigA transformed *E. coli* Rosetta2 (DE3) was used as a negative control in a simple batch purification of a culture induced at ∼0.6 OD600 nm for 3 h at 37°C. Briefly, 200 µl of Ni-NTA resin was incubated with expression lysate in an Eppendorf tube for 1 h at 4°C. The resin was washed 3 times with 500 µl of wash buffer containing 100 mM imidazole. The eluate was separated by Tricine-SDS–PAGE.

WhiB7–SigA_C82_ complexes were similarly isolated using the co-expression vector pR4B7. The absorbance at 280 nm was used to estimate the protein concentration with an absorbance of 1 corresponding to 0.93 mg/ml.

### Ultraviolet spectroscopy

A Hitachi U-3010 spectrophotometer was used to monitor absorbance with a scan rate of 300 nm/min. For the diamide-treated samples, diamide was added to a final concentration of 7 mM. Diamide interfered with measurements below roughly 380 nm.

### Minimal inhibitory concentration (MIC) determination

MIC determinations were carried out as previously described (10).

### Disk assay

*M. smegmatis *strains were grown in 3 ml of 7H9 for 2 days (i.e. to stationary phase). 7H9 agar (no tyloxapol) was prepared with a 15 ml of 1.5% (w/v) agar base and 7 ml of 0.5% (w/v) top agar. Once the base was solidified, the strains were inoculated to a final OD 600 nm of 0.005 into the 7 ml of top agar cooled to 50°C. The suspension was mixed and immediately poured onto the agar base. When the agar solidified, blank paper disks (BD 231039) were placed on the top agar, and 7.25 µl of antibiotic solution was spotted as indicated. The plates were then incubated in a closed non-airtight humid container at 37°C for 48 h and pictures taken.

## RESULTS

### WhiB7 binds the primary vegetative sigma factor SigA

Transcriptional activators that bind nucleotide sequences overlapping or adjacent to the −35 promoter hexamers generally increase the affinity or stabilize sigma factor binding to the promoter, thereby stimulating transcription (40). The −10 and −35 hexamers of the *whiB7* promoter and genes in its regulon are similar to the consensus sequence of promoters recognized by the primary vegetative sigma factor in *Mtb*, SigA (10). A conserved AT-rich motif, that may serve as the WhiB7-AT-hook binding site, is located immediately upstream of the −35 hexamer. This suggested a possible interaction between WhiB7 and SigA proteins. Indeed, WhiB3, a WhiB7 paralog, binds a 160 amino acid C-terminal fragment (residues 369–528) of SigA (referred to as SigA_C160_) (16). SigA_C160_ spans several SigA structural domains including the terminus of region 2, as well as region 3 and region 4 (the −35 hexamer binding domain) (41). Steyn *et al.* (16) showed that WhiB3 and SigA_C160_ are not able to interact when an amino acid located at the C-terminus of region 4.2 (residue 515) is mutated (R515H). We cloned a 170 amino acid C-terminal fragment (residues 297–466) of *M. smegmatis *SigA that was 99.6% identical to *M. tuberculosis *SigA (amino acids 359–528; referred to as SigA_C170_). An *E. coli* two-hybrid system (BacterioMatch II), as well as co-expression/pull-down experiments, were used to test the possible protein–protein interaction between *M. smegmatis* WhiB7 and SigA_C170_.

**Figure 2.**
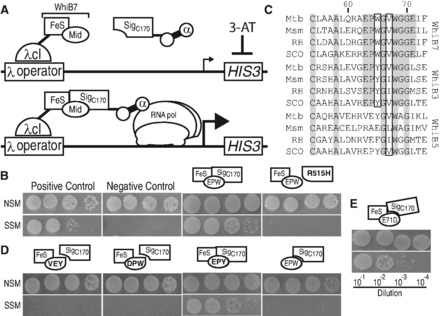
WhiB7ΔC19 binds to the C-terminus of SigA but not its R515H mutant. (**A**) Schematic description of the BacterioMatch II two-hybrid system. A bait protein fusion (*M. smegmatis* WhiB7 + λcI) is targeted to a weak promoter expressing *HIS3 *by λcI/λ-operator interaction. 3-amino-1,2,4-triazole (3-AT) inhibits HIS3 activity resulting in histidine auxotrophy. If the target protein fusion (*M. smegmatis* SigA + α) interacts with the bait protein, α promotes the recruitment of RNAP increasing the expression of *HIS3 *and restoring the ability of the cell to grow on media lacking histidine. (**B**) Spotted dilutions (10^−^^1^–10^−^^4^) of reporter co-transformants on non-selective and selective screening media (NSM and SSM, respectively) to test protein interaction. Protein–protein interaction is indicated by growth on SSM. Bait and target vectors (pBT/pTRG) without inserts served as a negative control. Positive control plasmids (pLGF2/pGAL11, top left) were from Strategene. Combinations of bait and target proteins tested are illustrated. WhiB7 is represented in two parts: the cysteine iron binding box ‘FeS’ (aa 1–54) and the glycine rich tryptophan turn region (oval ‘mid’; aa 55–80). The WhiB7 construct was partnered with a C-terminal fragment of SigA ‘SigA_C170_’, or its R515H mutant, ‘R515H’, as indicated. Results are representative of at least three independent co-transformants. (**C**) Peptide sequences of WhiB7 (top), WhiB3 (middle) and WhiB5 (bottom) spanning a region from the fourth cysteine to the first glycine of the G(V/I)WGG turn from *M. tuberculosis *(Mtb), *M. smegmatis* (Msm), *R. jostii *(RH) and *S. coelicolor *(SCO). Conserved residues are highlighted gray and the chemically similar residues are boxed. (**D** and **E**) Similarly to (B), SigA_C170_ was partnered with various WhiB7 bait mutants or a truncated construct. Mutations in the ‘mid’ region are bolded in the oval; (D) EPY = W65Y, VEY = E63V P64E W65Y, DPW = E63D, (E) E71D = E71D.

Two-hybrid systems are designed to monitor interactions between two proteins based on their abilities to localize RNAP to a promoter upstream of a reporter gene. In the BacterioMatch II system, this results in higher expression of a histidine biosynthetic enzyme (HIS3) that can be monitored in a histidine auxotrophic reporter strain of *E. coli* grown on selective screening media (SSM) in the presence of a HIS3 inhibitor (3-AT: Figure 2A). The strength of the interaction determines growth, measured as plating efficiency. To ensure that WhiB7 DNA-binding activity did not interfere with targeted localization to the reporter gene, the WhiB7 used as bait was truncated to remove the C-terminal AT-hook (WhiB7ΔC19; pBTW7ΔC19; Figure 1). Indeed, the use of WhiB7ΔC19 as bait and SigA_C170_ (pTRG170) as target allowed growth on SSM (Figure 2B). This provided the first evidence of WhiB7–SigA interaction. WhiB7ΔC19–SigA_C170_ interaction was prevented by the R515H mutation (Figure 2B) corresponding to the experiments done with WhiB3-SigA_C160_. Importantly, expression of neither bait nor target protein alone promoted growth on SSM (Supplementary Figure S1), and all strains grew normally on non-selective screening media lacking histidine without the HIS3 inhibitor (NSM; Figure 2B).

**Figure 3.**
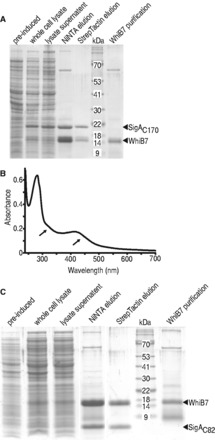
WhiB7 forms a stable and soluble complex with region 4 of SigA in *E. coli*. (**A**) A C-terminal fragment of SigA (SigA_C170_) was co-expressed with WhiB7 (whole-cell lysate). Soluble proteins (lysate supernatant) were passed through Ni-NTA resin allowing purification of His tagged-WhiB7 and its binding partner, SigA (NiNTA elution). The Ni-NTA eluate was then passed through StrepTactin resin to bind strepII tagged SigA resulting in co-purification of WhiB7 (StrepTacin elution). Protein molecular mass are estimated from standards with indicated masses (kDa). WhiB7 purification is the WhiB7 preparation used for *in vitro *run-off experiments. (**B**) Absorption of the StrepTactin eluate over a range of wavelengths. There are broad shoulders (arrows) between 300 and 350 nm and between 400 and 450 nm, which is indicative of FeS clusters. (**C**) Region 4 of SigA (SigA_C82_) was co-expressed with WhiB7 (whole cell lysate). Soluble proteins (lysate supernatant) were passed through Ni-NTA resin allowing purification of His tagged-WhiB7 and its binding partner SigA(region4) (NiNTA elution). The Ni-NTA eluate was then passed through StrepTactin resin to bind strepII tagged region 4 of SigA resulting in co-purification of WhiB7 (StrepTacin elution). WhiB7 purification is the WhiB7 preparation used for *in vitro *run-off experiments (nb, a degraded form of WhiB7 was present in the preparation).

To confirm WhiB7–SigA binding, *in vitro* pull-down experiments were done using full length WhiB7 fused to a 10xHis-tag (N-terminal) and SigA_C170_ fused to a strepII-tag (N-terminal). Proteins were co-expressed in *E. coli *(Figure 3A; lysate supernatant), and the soluble cytoplasmic fraction was passed through a Ni-NTA column that bound 10xHis-WhiB7. Elution from the column with imidazole co-purified SigA_C170_ along with WhiB7 (Figure 3A; NiNTA elution). The eluate was then passed through a Strep-Tactin column that bound strepII-tagged SigA_C170_. Elution from StrepTactin (using desthiobiotin) once again co-purified SigA_C170_ along with WhiB7 (Figure 3A; StrepTactin elution), demonstrating that WhiB7 and SigA formed stable and soluble complexes. The fact that SigA_C170_ was not purified on the Ni-NTA column in the absence of WhiB7 (Supplementary Figure S2) demonstrated specificity. The ultraviolet spectrum of the purified WhiB7–SigA_C170_ complex, which eluted as a brown/yellow solution, contained broad shoulders between 300–350 and 400–450 nm indicating the presence of FeS bound holo-WhiB7 (Figure 3B). Based on the extinction coefficient of iron within bound FeS clusters at 400 nm [estimated to be 4000 M^−^^1^cm^−^^1^ (42)], the spectroscopic data indicated an iron to WhiB7–SigA complex ratio of 1.91 (±0.05) to 1.

The SigA_C170 _fragment used in these experiments extended from the end of region 2 through regions 3 and 4. As the R515H mutation that prevents WhiB7–SigA interaction was located at the end of region 4.2, we investigated whether region 2 and 3 in SigA_C170_ were dispensable for interaction with WhiB7. To map the WhiB7 binding site more precisely, we constructed a vector expressing the C-terminal 82 amino acids of SigA including only region 4 (SigA_C82_; pR4B7). As was found with the SigA_C170_ fragment, WhiB7 co-purified with SigA_C82_ (Figure 3C). We conclude that the WhiB7 binding site is localized in region 4 (most likely region 4.2) of SigA.

### Regions and residues of WhiB7 that are essential for SigA binding

WhiB7 is composed of three structural motifs predicted by its amino acid sequence, including the FeS cluster binding domain, middle domain and AT-hook domain (Figure 1). The AT-hook motif located at its C-terminus likely interacts with the conserved AT-rich region upstream of its promoter (10) and was not required for SigA binding (Figure 2B). This indicated that the residue(s) responsible for SigA interaction were within the FeS cluster binding domain and/or the middle domain.

Predictions of conserved Wbl structural features by Soliveri *et al. *(7) suggest that a loop between the FeS cluster domain and the G(V/I)WGG (glycine turn motif located at the core of the middle domain) ‘is a prime candidate for an interaction with another conserved cellular component (perhaps RNAP, bound adjacent to a Wbl protein at a promoter)’. As WhiB3 interacts with SigA (16), but WhiB5 does not (11), we compared WhiB7, WhiB3 and WhiB5 loop sequences from several actinomycetes genera, including *Mycobacterium*, *Streptomyces *and *Rhodococcus*. Both WhiB7 and WhiB3 proteins contained a similar triplet motif, EPW and EPY, in all species, whereas WhiB5 had various dissimilar sequences (Figure 2C). Numerous transcriptional regulators are known to bind region 4 of SigA, with interactions typically occurring between basic SigA amino acids (i.e. arginine or lysine) and acidic amino acids (i.e. glutamate or aspartate) in the activator protein (43). Therefore, our alignment suggested that the conserved anionic glutamate in WhiB7 (E63) and WhiB3 might interact with cationic arginine (R515) of SigA. The WhiB7 bait for the BacterioMatch system, WhiB7 (EPW), was therefore mutated to contain sequences mimicking WhiB3 (EPY), or WhiB5 (VEY). One other construct was made in which the glutamate was mutated to a chemically similar residue aspartate (DPW). Neither WhiB7 bait in which EPW was mutated to the WhiB5 sequence (pBTW7epy) nor the construct containing the glutamate to aspartate mutation (pBTW7d) interacted with SigA strongly enough to promote growth, whereas mutation to the WhiB3 sequence (pBTW7vey) retained activity (Figure 2D). This suggested that the glutamate was essential for SigA interaction and that WhiB7 and WhiB3 may bind SigA in the same manner.

A fragment of WhiB7 containing the EPW motif, spanning the region from the last cysteine to the border of the AT-hook domain (the middle domain of WhiB7 diagrammed in Figure 1), was then tested for SigA binding (pBTW7mid) using the two-hybrid system. This fragment was inactive (Figure 2D). As WhiB7 bound to SigA contained an FeS cluster (Figure 3B), the result may indicate that tertiary structural features such as FeS cluster binding may stabilize SigA binding.

Mutations of the conserved cysteines to alanines in WhiB1 or WhiBTM4 eliminates FeS cluster binding (21,44), and at least two cysteine to alanine mutants of WhiB2 are inactive *in vivo* (26). Therefore, to investigate whether the FeS cluster contributed to WhiB7 binding of SigA, the third cysteine (pBTW748) and both the second and third cysteines (pBTW74548; the CXXC motif), were mutated to alanines (Figure 1) and tested using the BacterioMatch system. Neither construct promoted growth under selective conditions, indicating that the complex between SigA and WhiB7 was absent (Supplementary Figure S3A). To further investigate these results using pull-down experiments, mutant genes were cloned into the expression vector used to co-express WhiB7 and SigA. Surprisingly, neither mutant protein was stably expressed either alone (Supplementary Figure S3B) or with SigA (Supplementary Figure S3C). These results suggested that, unlike WhiB1, loading of the FeS cluster was essential for WhiB7 stability in *E. coli*. To ensure that the other mutants analyzed using the BacterioMatch system (Figure 2D) were stable, each was expressed alone and could be purified at comparable levels to wild-type WhiB7 (Supplementary Figure S4). This demonstrated that WhiB7 instability was a unique function of the Cys to Ala mutations, presumably due to loss of FeS cluster binding or perhaps improper folding of its precursor apo protein.

### WhiB7 is a redox sensitive transcriptional activator

**Figure 4.**
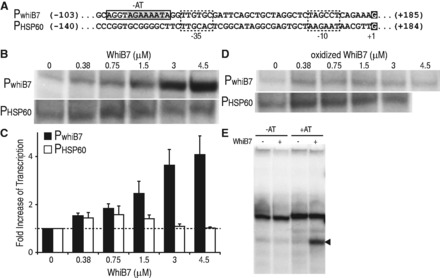
*In vitro *run-off analysis shows WhiB7 is an autoregulatory, redox-sensitive transcriptional activator targeting a conserved AT rich region upstream of its promoter. (**A**) Sequence of the *whiB7 *(P_whiB7_) and HSP60 (P_HSP60_) promoters. The potential WhiB7 binding site is highlighted in gray with region deleted for experiments in ‘E’ boxed. The −10 and −35 hexamers are boxed within dashed lines and the transcriptional start site is highlighted as white text on a black background. (**B**) Transcriptional products from the *whiB7 *(P_whiB7_) and HSP60 (P_HSP60_) promoters with the addition of an increasing amount of WhiB7 (0–4.5 µM). (**C**) Quantification of transcriptional activity observed in ‘B’, as fold increase versus no WhiB7 of the *whiB7 *(black) and HSP60 (white) promoters. Transcripts from three reactions were quantified and averaged. Error bars represent standard deviation. (**D**) Transcriptional products from the *whiB7 *(P_whiB7_) and HSP60 (P_HSP60_) promoters with the addition of an increasing amount of oxidized WhiB7 (0–4.5 µM). Owing to the staggered nature of preparing multiple run-off reactions in parallel, the diamide pre-treatment time of WhiB7 ranged from 15 (lowest diamide concentration) to 26 min (highest diamide concentration). (**E**) Transcriptional product (arrow) of the *whiB7 *promoter with (right) and without (left) the conserved at AT-rich region (see ‘A’). Reactions were carried out in the presence (+; 3.0 µM) or absence (−) of WhiB7. The other prominent bands represent non-specific end-to-end transcripts.

The *whiB7* promoter contains a conserved AT-rich region, which may serve as a WhiB7 binding site (10). To test whether WhiB7 directly catalyzed transcription of its promoter, WhiB7 was purified for transcriptional run-off assays. WhiB7 contains an FeS cluster that is sensitive to air but can be significantly stabilized by a reductant (20). The 10xHis-WhiB7 was purified at 4°C under aerobic conditions using a Ni-NTA column. It eluted as a brown solution, characteristic of proteins binding an FeS cluster. The ultraviolet spectrum contained broad shoulders between 300–350 and 400–450 nm with a characteristic shape indicative of [2Fe2S] clusters (Figure S5). Although anaerobic reconstitution allows other WhiB holo proteins to co-ordinate a [4Fe-4S] cluster, aerobic purification of those proteins also yields the more oxygen stable [2Fe-2S] form (20). As the purification of WhiB7 was done aerobically, the eluate was expected to contain both apo- and holo- WhiB7 in various redox states. To stabilize FeS clusters, purified WhiB7 was supplemented with DTT and flash frozen. Holo-RNAP, loaded with a mixture of sigma factors, was isolated from exponentially growing *M. smegmatis. *RNAP activity was assayed using the *whiB7* and HSP60 promoters (Figure 4A) in the presence of increasing concentrations of WhiB7. Transcriptional run-off products were analyzed after separation on denaturing polyacrylamide gels (Figure 4B). WhiB7 catalyzed up to a 4-fold increase in transcription of its own promoter in a concentration-dependent manner and had no significant effect on transcription from the HSP60 (Figure 4C). There was a relatively small increase of HSP60 activity when lower concentrations of WhiB7 were added. Although we cannot rule out a minor direct effect, the fact that higher concentrations did not promote larger transcription increases from the HSP60 promoter indicates that it is insignificant. Control experiments using truncations of adjacent upstream and downstream regions of the *whiB7* promoter confirmed our *in vivo* mapping (10) of the start site and direction of transcription (Supplementary Figure S6).

Studies of growing cultures had previously established that reducing conditions generated by supplementing the medium with DTT synergistically enhanced the activation of the *whiB7* promoter by erythromycin, whereas oxidizing conditions (supplementing with diamide) reduced activation (10). Transcriptional run-off experiments were therefore carried out to determine whether this was a direct effect of diamide on WhiB7. In studies of other holo WhiB proteins, diamide is known to release their FeS clusters generating oxidized apo proteins with intramolecular disulphide bonds between the conserved cysteines (9,15,20). Although the presence of diamide interfered with absorbance readings below ca. 400 nm, a clear, time-dependent drop in the 450 nm shoulder was observed indicating partial loss of the FeS cluster (Supplementary Figure S5B) (42). Consistent with our *in vivo *observations, incubation of WhiB7 with diamide eliminated its ability to activate transcription (Figure 4D). Furthermore, the observation that diamide did not inhibit transcription from the WhiB7-independent HSP60 promoter demonstrated specificity for WhiB7, i.e. it was not due to oxidation of other components in the run-off reaction mix.

The AT-rich region upstream of the *whiB7* promoter is required for WhiB7-mediated transcriptional activation *in vivo *(10). When this AT-rich region was deleted, *in vitro *transcription was no longer promoted by WhiB7, confirming its requirement for WhiB7-dependent transcription activation (Figure 4E).

### The role of WhiB7 in providing intrinsic drug resistance is dependent on its interactions with a promoter sequence motif and SigA

Having established the requirement of the AT-rich sequence upstream of the −35 hexamer for WhiB7-catalyzed transcription (Figure 4), experiments were carried out to investigate the role of the WhiB7 C-terminal AT-hook motif in targeting this region *in vivo*. A C-terminally truncated protein lacking the AT-hook was tested for its ability to complement the *M. smegmatis whiB7* knock out (KO) strain. Resistance to representative members of several structural classes of antibiotics within the *whiB7* sensitivity spectrum were screened, including an aminoglycoside (spectinomycin), a tetracycline (tetracycline) and macrolides (clarithromycin and roxithromycin). The *whiB7* KO strain was more sensitive to spectinomycin (8–16-fold), tetracycline (8-fold), clarithromycin (>32-fold) and roxithromycin (>32-fold) (Table 2). Constitutive expression of full-length WhiB7 (pFB7), but not a C-terminally truncated WhiB7ΔC19 (pFB7AT), restored antibiotic resistance in the *M. smegmatis whiB7* KO, demonstrating that the AT-hook was required for activation of resistance systems (Table 2). Interestingly, expression of WhiB7ΔC19 in the parental background consistently lowered its antibiotic resistance 2-fold (relative to the vector control and pFB7), suggesting that the WhiB7ΔC19 may compete with functional WhiB7 protein for SigA binding (Table 2) to sequester it in an inactive form. In addition, an N- and C-terminally truncated WhiB7 (WhiB7ΔN19C6; pB7fun), which corresponded to the conserved ‘functional’ region of WhiB7 proteins (Figure 1) restored antibiotic resistance (Table 2). This indicated that the N- and C-termini do not contribute to WhiB7’s antibiotic resistance function.

**Table 2.** Minimum inhibitory concentrations of *M. smegmatis* and *whiB7 *mutant (KO) expressing WhiB7 and WhiB7ΔC19, as well as *M. smegmatis* FB7

Strain:	Minimum inhibitory concentration (µg/ml)^a^ *M. smegmatis* mc^2^155							
	Parental			*whiB7* KO				
Vector:	pMV261	pFB7	pFB7AT	pMV261	pFB7	pFB7AT	pFB7d	pB7fun
Spectinomycin	80–40	80	40	5	80	2.5	10	160–80
Tetracycline	1	1	0.5	0.13	1	0.063–0.03	0.25–0.13	1
Clarithromycin	3	3	1.5	≤0.094	3	≤0.094	0.094	6
Roxithromycin	24	24	12	≤0.38	24	≤0.38	≤0.38	24

^a^MIC ranges represent three independent transformants.

**Figure 5.**
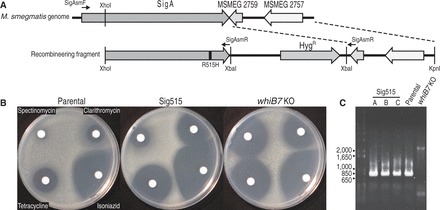
The SigA R515H mutant mimics the *whiB7 *mutant (KO) multi-drug susceptible phenotype. (**A**) Outline of the recombineering strategy used to construct *M. smegmatis* Sig515. A double recombination event replaced a region of the SigA gene (top) with a mutated allele R515H (bottom). Positive selection for insertion was provided by the hygromycin resistance gene (Hyg^R^). The primers SigAsmF and SigAsmR, used to amplify *sigA *for sequencing to confirm insertion of the mutation, are indicated. (**B**) Disk assay for the resistance of *M. smegmatis* Parental (left), Sig515 (middle) and *whiB7 *KO (right). Total micrograms spotted were spectinomycin 145, clarithromycin 2.9, isoniazid 145 and tetracycline 0.7. The results are representative of three independent Sig515 recombinants or the other strains performed in triplicate. (**C**) PCR products of the amplification of *whiB7 *and its promoter from *M. smegmatis* parental, the three Sig515 recombinants, and *whiB7 *KO. All strains, with the exception of *whiB7 *KO, had the expected ca. 800 bp product. Sizes of the ladder (left) are indicated.

In *Mtb*, the SigA R515H mutant is less virulent, mimicking the phenotype of a mutation of its paralog, WhiB3 (15,45). Binding of WhiB7 and SigA was similarly prevented by the SigA R515H or WhiB7 E63D mutations (Figure 2), predicting that these mutations might generate a multi-drug sensitivity phenotype found in the *whiB7* mutant. We replaced wild-type *sigA *with an R515H mutant allele to construct *M. smegmatis *Sig515 (Figure 5A). Although levels of resistance to representative antibiotics not in the *whiB7* sensitivity spectrum (including danofloxacin and isoniazid) were not significantly changed (Table 3), the mutant was at least 4-fold more sensitive to antibiotics within the *whiB7* sensitivity spectrum (including tetracycline, spectinomycin and clarithromycin) (Table 3). Interestingly, the decrease in sensitivity to spectinomycin was affected less by the SigA R515H mutation than by the *whiB7* deletion. Disk assays were done to further evaluate resistance levels (Figure 5B). The Sig515 mutant was slightly more resistant to clarithromycin and tetracycline than the *whiB7* KO mutant, but was much more resistant to spectinomycin (Table 3). To ensure that *whiB7* was not disrupted in the Sig515 strain, the presence of the gene and its promoter were confirmed by PCR (Figure 5C).

**Table 3.** Comparison of drug susceptibility of *M. smegmatis* parental, Sig515 and *whiB7 *mutant (KO) in liquid and on solid media

Antibiotic	*M. smegmatis* mc^2^155					
	MIC (µg/ml)^a^			Diameter of inhibition zone (mm)^b^		
	Parental	Sig515	*whiB7* KO	Parental	Sig515	*whiB7* KO
Spectinomycin	80	20	10	0	18.1 ± 2.6	34.3 ± 1.3
Clarithromycin	1.5	0.19–0.09	0.094	15.3 ± 0.3	27.5 ± 1.2	30.3 ± 0.6
Tetracycline	2	0.5–0.25	0.5	21	31.9 ± 0.9	35.3 ± 0.3
Isoniazid	16	8	16	48	49.8 ± 0.4	46.7 ± 1.2
Danofloxacin	0.35	0.35	0.35			

^a^MICs represent the range of three biologically independent replicates for parental and *whiB7* KO strains. They are the range of three independent Sig515 mutants.

^b^Diameters are the average of triplicate experiments for parental and *whiB7* KO strains. For Sig515, they are the average of three independent recombinants performed in duplicate. If measurements showed variation the standard deviation is indicted as ‘±’. Total µg spotted: spectinomycin 145, clarithromycin 2.9, tetracycline 0.725, isoniazid 145.

Finally, we tested the ability of WhiB7 E63D (pFB7d) to complement the *whiB7* KO mutation. Similar to the SigA R515H mutation, the complemented strain showed a *whiB7* specific multi-drug susceptibility profile (Table 2) confirming that WhiB7 E63D did not activate a drug resistance response.

### Modeling WhiB7 and its interaction with SigA and DNA

**Figure 6.**
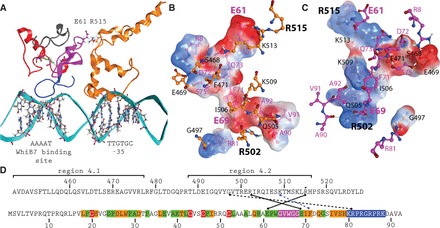
Structural prediction of WhiB7 and its interaction with SigA/DNA. (**A**) A structural model involving *Mtb* WhiB7, *Mtb* SigA and DNA. WhiB7 is shown as a ribbon with the three functional regions highlighted; variable N-terminus (gray), iron sulphur cluster binding domain (red), middle domain that interacts with SigA (pink) and DNA-binding domain (blue) positioned in the DNA minor groove. The homology model of *Mtb *SigA region 4 [based on *E. coli* SigA *E. coli* (PDB: 4IGC_X)] is shown as an orange ribbon, interacting with the DNA at the −35 hexamer position. Interaction surfaces of (**B**) WhiB7 as viewed by SigA (interactive SigA residues are superimposed) and (**C**) SigA as viewed by WhiB7 (interactive WhiB7 residues are superimposed). The electrostatic surfaces of interacting residues are shown (blue: positively charged, red: negatively charged). Hydrogen and ionic bonds are displayed as green lines. For clarity, hydrogen atoms not involved in interactions are hidden. Colors of amino acids modeled correspond to those in (A). WhiB7 and SigA residues are indicated as pink and black font, respectively. Experimentally verified interactions are labeled with larger font. (**D**) Potential conserved interactions between WhiB7 (below) and region 4 of SigA (top). The experimentally determined point of interaction (R515) is starred. The *Mtb *WhiB7 maintains the colouring from Figure 1 to highlight conserved residues. Ionic bonds are indicated by solid lines, hydrogen bonds as dashed lines, and hydrophobic interactions as dashed blue lines.

Although Wbl proteins have attracted the attention of many laboratories, tertiary structural data are not available for any of these proteins. This is generally attributed to structure predictions that their peptide sequences are primarily disordered and the fact that FeS-containing regulatory proteins are notoriously unstable (46). WhiB7 in *Mtb *is a 92 amino acid protein (UniProt accession number: Q6MX01) that does not have sufficient sequence similarity with any known structures for homology modeling or structure-sequence threading. Therefore, a 3D structural model of WhiB7 was built using a *de novo* prediction method as implemented in the Robetta server (47). The top 10 WhiB7 models were examined for positioning of the four cysteine residues to coordinate a [2Fe-2S] FeS cluster, and a C-terminal loop resembling an AT-hook (48). Our experimental data predicted an interaction between E61 of WhiB7 and R515 of SigA. Using this information, a homology model of SigA region 4 of *M. tuberculosis* (UniProt accession number: P0A602) was built using MODELLER (49) based on the *E. coli *SigA structure [PDB: 4IGC_X; (50)]. Region 4 was 60% similar in *E. coli *and *Mtb* sequences. The potential interacting poses between WhiB7 and SigA were then evaluated by using protein–protein docking predictions from the ClusPro 2.0 server (51) and the E61–R515 interaction shown experimentally (Figure 2). To further define DNA-binding residues, the most likely protein docking model (best of 12 candidates) was superimposed with the RNAP holoenzyme-DNA complex [PDB: 1L9Z; (52)]. Using the positions of expected binding sequences for WhiB7 and SigA (Figure 4A), the model of the complex was energy minimized in the AMBER99 force field. Figure 6A illustrates the overall structural model of WhiB7, SigA and DNA. The predicted interface between WhiB7 and SigA based on protein–protein docking was further examined for interacting residues. Remarkably, the electrostatic surfaces of WhiB7 (Figure 6B) and SigA (Figure 6C) had complementary charges and shapes. In addition to the E61 and R515 interaction, there were numerous potential close (within 4.5 Å) interactions (Supplementary Table S2) between WhiB7 and SigA that were identified using the Molecular Operating Environment suite. Interactive residues corresponding to conserved WhiB7 amino acids are diagramed in Figure 6D. Interestingly, a glutamate residue downstream of the tryptophan turn, E69 (E71 in *M. smegmatis*) (Figure 2C), was identified as a partner for a second potential ionic interaction with SigA. To provide evidence for the validity of the model, E71 in *M. smegmatis* was mutated to an aspartate (pBTW71d) and tested by the two-hybrid assay. Unlike the E63D mutant, the E71D mutant in combination with SigAC_170_ promoted growth under selective conditions, although not as strongly as the wild-type or W65Y mutant (Figure 2E). The decreased growth (corresponding to a 10-fold dilution) suggested that WhiB7–SigA interaction was weakened by the E71D mutation, but not as effectively as the E63D mutation. Thus, the model correctly identified R71 as a residue contributing to SigA interaction.

## DISCUSSION

In *Mtb*, seven *whiB *genes are implicated in a variety of fundamental metabolic processes. Whether the corresponding gene products act exclusively as transcriptional regulators and/or have additional functions as thioredoxins or chaperones continues to be debated. Our *in vitro* run-off experiments provided the first direct biochemical proof of transcriptional activation by a WhiB protein. We also dissected WhiB7’s predicted functional domain (Figure 1A), demonstrating three sequence motifs that contribute to protein stability (FeS cluster binding domain), binding of SigA (middle domain) and targeting RNAP to a specific subset of vegetative promoters (DNA-binding domain).

Our experiments showed that WhiB7 is a redox-sensitive transcriptional activator *in vitro*, thereby proving a direct role in transcriptional activation implied by previous genetic studies (2,10). The *in vitro* data support *in vivo* work showing promoter specificity requires an AT-rich sequence upstream of the *whiB7* promoter, and sensitivity of WhiB7 function to redox conditions.

Isolation and sequencing of Wbl proteins predicted that positively charged amino acids within their C-terminal regions determine DNA-binding specificity (7). Mutations in homologous residues impair DNA binding activities of some mycobacterial WhiB proteins as well as their functions *in vivo* (26,44). Our studies of WhiB7 showed that removal of its C-terminus, containing an AT-hook motif, prevented activation of *whiB7-*specific antibiotic resistance *in vivo *(Table 2). Evidence for interaction between the AT-hook and the *whiB7* promoter was provided by experiments showing that deletion of a conserved AT-rich region immediately upstream of the *whiB7* promoter also prevented WhiB7-mediated transcriptional activation *in vivo* (10) or *in vitro* (Figure 4). There is little information about AT-hook containing proteins in prokaryotes, but in eukaryotes the motif is found in a variety of DNA-binding regulatory proteins (53). The AT-hook binds the minor groove of AT-rich DNA (22) rather than specific sequences recognized by many other DNA binding domains. It is often found on proteins containing additional DNA-binding modules, suggesting that their AT hooks alter their affinity or specificity (53). The *whiB7* promoter (Figure 4A) strongly resembles the consensus sequence of SigA-targeted promoters, and the proximity of the AT-hook binding motif to the −35 hexamer suggested that WhiB7 might act by stabilizing sigma factor–DNA binding (10,40,43). WhiB7 bound to SigA would add the AT-hook to the repertoire of SigA DNA binding modules, providing additional specificity for promoters containing AT-rich regions upstream of their −35 hexamers. In essence, the AT rich region recognized by WhiB7 would act as a discriminator similar to *cis-*acting promoter features that enhance sigma factor selectivity for distinct promoters (54).

Our experiments proved that WhiB7 (with or without the AT-hook) binds to the C-terminus (region 4) of SigA (Figure 2; Figure 3). The interaction was eliminated by specific mutations in region 4.2 of SigA (R515H) (Figure 2B). Steyn and collaborators have reported that the SigA R515H mutation prevented interaction between SigA and WhiB3 (16) and had a decreased virulence phenotype corresponding to that of a *whiB3* mutant (15,45). Our data demonstrated that the SigA R515H mutation also prevented interaction with WhiB7 and caused WhiB7-specific multi-drug susceptibility *in vivo *(Figure 5; Table 3). The fact that the SigA R515H mutant had a slightly different antibiotic sensitivity profile may indicate that R515 interacts with other proteins (including WhiB3) that have roles in intrinsic resistance.

Region 4.2 of SigA binds a variety of transcriptional activators, generally mediated by interactions between its positively charged residues and negatively charged residues of a regulatory partner protein (43). We found that E63 of WhiB7 was an essential residue for WhiB7–SigA binding (Figure 2) and is required for antibiotic resistance *in vivo *(Table 2). Importantly, this glutamate, along with adjacent amino acids (proline and tryptophan), were conserved across WhiB7 orthologs and similar in WhiB3 (Figure 2D). This implied that WhiB7 and WhiB3 bind SigA in a similar manner. Although conservation of this tripeptide motif was necessary for SigA binding, the middle domain alone was unable to bind (Figure 2D). This suggested additional sequence requirements that must be located upstream since the C-terminal DNA binding domain was dispensable. We conclude that sequences within the N-terminal domain encoding FeS cluster binding residues either interact directly with SigA or are needed for proper folding of regions within the middle domain that interact with SigA.

Wbl homologs contain four conserved cysteines at their N-termini that typically co-ordinate an FeS cluster. Alterations of FeS clusters, either conversion from [4Fe-4S] to [2Fe-2S], change of their redox states, or ejection of FeS clusters, are all mechanism of activation for FeS-containing transcriptional regulators (55). The event alters tertiary structure to either activate or restrict transcriptional activity of the protein. This versatility allows the evolution of FeS cluster-containing proteins able to respond to redox signals of different strengths. WhiB7–SigA complexes contained FeS clusters (Figure 3B) that may provide essential tertiary stability allowing the middle domain of WhiB7 to interact with SigA, thereby promoting transcription. Importantly, run-off transcription catalyzed by WhiB7 was inactivated by oxidation with diamide at concentrations that did not affect transcription of P_HSP60_, a standard vegetative promoter (Figure 4). This is consistent with *in vivo* studies demonstrating that diamide inhibits activation of the *whiB7* promoter by antibiotic treatment. Diamide is a thiol-specific reagent that oxidizes cysteines thereby releasing FeS clusters, typically leading to intramolecular disulphide bonds in the apo protein (9,15,20). WhiB7 cysteine mutants could not be stably expressed in *E. coli*, suggesting the protein would be unable to establish a stabilizing interaction with SigA (Supplementary Figure S3). Importantly, erythromycin treatment of *M. smegmatis *generates a highly reduced cytoplasm (10) that might favour holo-WhiB7 and promote WhiB7-mediated transcriptional activation. The fact that this reductive shift is dependent on *whiB7* (10) suggests that WhiB7 action may be autocatalytic, activating resistance systems and thereby generating a highly reducing environment to increase its activity.

WhiB7’s link to reducing conditions and stable loading of an FeS cluster is important for its role as a resistance gene and highlights the emerging theme that antibiotic-induced effects extend far beyond inhibition of single targets and resonate through cell metabolism, often involving redox perturbations. For example, production of the reductant H_2_S provides resistance to many structurally and functionally diverse antibiotics (56). In mycobacteria, mycothiol, the major thiol protectant, plays a role in resistance to oxidative stress and some antibiotics (57). Therefore, generation of WhiB7-mediated reducing potential may provide resistance to antibiotics by promoting WhiB7 transcriptional activation of discrete resistance genes and/or metabolic systems. Previous microarray data (2) did not indicate *whiB7*-dependent transcriptional changes of genes in the mycothiol biosynthesis pathway, suggesting the reductive shift may be due to an another WhiB7 activity, perhaps acting as a thioredoxin or activating other thiol reductant systems (20). Over-expression of WhiB7 in *Mtb *activates its regulon in the absence of antibiotics (2). This implies that WhiB7 only has to be expressed and loaded with an FeS cluster to function, which correlates with our *in vitro *run-off data. Once induced, expression of WhiB7 generates a resistance state that is not tailored to the inducing antibiotic. Instead, it provides broad spectrum resistance reflecting a generalized metabolic shift (10,35,58). Transcriptional activation of different *wbl* genes is induced by a variety of different conditions *in vivo*, presumably corresponding to the different metabolic functions of the Wbl proteins.

Underlying their wide variety of functions, each Wbl protein has unique partnerships with sigma factors and regulatory functions, which may be modulated by multiple redox states. Although comparisons of redox sensitive Wbl proteins using different genetic and biochemical assays is often challenging, conserved themes of their transcriptional regulatory activities have emerged. *Mtb* and *M. smegmatis* SigA R515H mutants are viable, suggesting that essential WhiB proteins (WhiB1 and WhiB2) do not interact with SigA in the same way as WhiB7 and WhiB3, or that they partner with other sigma factors. WhiB5 does not interact with SigA in a two-hybrid assay (11). By analogy to our *in vitro* run-off with WhiB7, WhiB3 probably activates promoters directly via similar, redox-sensitive interactions with SigA. However, both oxidized and reduced forms of holo-WhiB3 and the oxidized form of apo-WhiB3 bind DNA with different affinities (15). It is still unclear in which state WhiB3 might promote transcription (15). apo-WhiB1 represses transcription *in vitro* and therefore may not depend on interactions with a sigma factor (9,44). Overall, it is clear that SigA binding is not a universal feature of Wbl proteins, and that individual Wbls may have unique redox-sensitive interactions with different sites within SigA or alternative sigma factors. This is supported by the observation that four of the 12 *S. coelicolor wbl *genes are located proximal to genes encoding sigma factors that may serve as their binding partners (59); one plasmid-encoded Wbl protein is translationally fused to a sigma factor (60). Many of the questions raised by these data could be clarified by analysis of tertiary structure.

We took advantage of our experimentally determined constraints and the crystal structure of the *E. coli* SigA ortholog in *E. coli* to generate a model of WhiB7 and its interactions with SigA and DNA (Figure 6A). The model of the protein complex predicted that WhiB7 (Figure 6B) has a distinct negatively charged face that fits remarkably well with the positively charged face of SigA region 4 (Figure 6B). In further support of the model, a predicted glutamate (E71)—arginine (R502) interaction (Figure 6C) was confirmed to play a role in WhiB7–SigA binding (Figure 2E). Interestingly, the glutamate responsible for this additional ionic interaction is not conserved in WhiB3 (Figure 2C); evidence that other residues that are not conserved in WhiB7 and WhiB3 may determine unique interactions with SigA. This may lead to varied SigA affinity, which could impact WhiB7 or WhiB3 biological activity. Overall, the model of WhiB7–SigA interaction predicts that WhiB7’s AT-hook is in both the proper orientation and distance for DNA binding, allowing it to trigger transcriptional activation. Future biochemical and genetic dissection of WhiB7–SigA affinity will provide additional insight into how WhiB7 promotes transcription.

In summary, this work has defined three distinct WhiB7 domains that function interactively to form a redox-sensitive transcriptional activator of intrinsic drug resistance genes in *Mtb* (Figure 1). The FeS cluster-binding domain likely stabilizes the tertiary structure of the holo-protein, allowing the middle domain to bind SigA. The AT-hook of WhiB7 allows the RNAP complex to target and specifically increase the expression of a family of promoters (the WhiB7 regulon) that contain an AT-rich region shortly upstream of their −35 hexamers. By analogy to genetic studies, chemical inhibition of WhiB7’s AT-hook- DNA or WhiB7–SigA interaction would prevent WhiB7 function and lead to multi-drug susceptibility. The fact that WhiB7 and WhiB3 bind to the same region of SigA to target genes in their respective regulons suggests that an inhibitor could prevent binding of both WhiB7 and WhiB3, thereby increasing susceptibility to diverse antibiotics as well as decreasing virulence. The C-terminal 33 amino acids of *Mtb *SigA are highly conserved across the Actinomycetes taxon, indicating that the molecular mechanism of WhiB7 and WhiB3 action may also be conserved. Therefore, inhibitors of WhiB7 or WhiB3 interactions with SigA could likely be applied to pathogens in related genera including *Corynebacterium* and *Nocardia*. Future characterization of these important proteins will not only provide strategies to understand intrinsic drug resistance but also generate important insights into how *Actinomycetes* integrate their physiology, division and differentiation programs.

## SUPPLEMENTARY DATA

Supplementary Data are available at NAR Online, including [61].

## FUNDING

The Canadian Institute of Health Research [MOP-82855 to C.J.T.]; British Columbia Lung Association (to C.J.T.); Natural Sciences and Engineering Research Council of Canada (to G.B.S.). Funding for open access charge: Canadian Institute of Health Research.

*Conflict of interest statement*. None declared.

## Supplementary Material

Supplementary Data
