# Supplementary material for: The mycobacterial antibiotic resistance determinant WhiB7 acts as a transcriptional activator by binding the primary sigma factor SigA (RpoV)
Source: Nucleic Acids Res. 2013 Aug 28;41(22):10062–76. doi: 10.1093/nar/gkt751 (PMC3905903; doi:10.1093/nar/gkt751)
Supplement: Supplementary Data [file supp_41_22_10062__index.html]

The mycobacterial antibiotic resistance determinant WhiB7 acts as a transcriptional activator by binding the primary sigma factor SigA (RpoV) — The mycobacterial antibiotic resistance determinant WhiB7 acts as a transcriptional activator by binding the primary sigma factor SigA (RpoV) — Supplementary Data 

# The mycobacterial antibiotic resistance determinant WhiB7 acts as a transcriptional activator by binding the primary sigma factor SigA (RpoV)

## Supplementary Data

files

**Files in this Data Supplement:**

- Supplementary Data - pdf file
