# Supplementary material for: The mycobacterial antibiotic resistance determinant WhiB7 acts as a transcriptional activator by binding the primary sigma factor SigA (RpoV)
Source: Nucleic Acids Res. 2013 Aug 28;41(22):10062–76. doi: 10.1093/nar/gkt751 (PMC3905903; doi:10.1093/nar/gkt751)
Supplement: Supplementary Data [file supp_gkt751_nar-01852-h-2013-File008.pdf]

SUPPLEMENTARY DATA

Supplemental Materials and Methods

Construction of constitutively expressing *whiB7* vectors

The upstream region of *whiB7* was amplified from the *M. smegmatis* genome by PCR using the primers FB7L_F and FB7L_R. *whiB7* and its downstream region were amplified using FB7R_F and FB7R_R adding an N- terminal 3xFLAG tag. The upstream region was digested with KpnI/ NcoI, the downstream region with NcoI/ HindIII and a three-way ligation was performed with KpnI/ HindIII digested pUC19 to construct pFB7SM. 3xFLAG::*whiB7* was then amplified by PCR from pFB7SM using the primer FlagB7smF with either FlagB7smR for full length *whiB7* or FlagB7smR_AT for a shortened *whiB7* lacking the C-terminal AT-hook (ΔC19). The *groEL2* promoter (HSP60) was amplified by PCR from pMV261 using the primers HSP60F and HSP60R_pst. The HSP60 amplicon was digested with XbaI/ PstI while the *whiB7* amplicons with PstI/ HindIII. The fragments were combined into XbaI/ HindIII digested pMV261 to construct the *whiB7* constitutively expressing pFB7 and AT-hookless *whiB7* expressing pFB7AT. This enured the expression of the constructs without the N-terminal GroEL2 amino acids encoded upstream of the usual pMV261 multiple cloning site. Both constructs contained a N-terminal 3xFLAG-tag for future immunoprecipitation experiments. The QuickChange Lightning mutagenesis kit (Stratagene) was used according to manufacturer instructions to mutate pFB7 to express WhiB7 E63D using the primers B7_D_F and B7_D_R, generating pFB7d. The functional region of WhiB7 (WhiB7ΔN19C6; Figure 1) was amplified from the *M. smegmatis* genome by PCR using the primers B7fun_F and B7fun_R. The amplicon was digested with NcoI and then blunted using NEB's Quick blunting kit. The amplicon was then digested with HindIII. Similarly, pFB7 was digested with PstI and blunted, followed by digestion with HindIII. The vector backbone fragment was ligated with the digested B7fun amplicon to generate the constitutively expressing *whiB7*ΔN19C6 vector pB7fun. Each of the vectors was then transformed into *M. smegmatis whiB7* KO; transformants were assayed for restoration of antibiotic resistance.

Construction of 10xHis:WhiB7 expression vector

whiB7 was PCR amplified from the *M. smegmatis* genome using the SRG-15 and SRG-16 primers. The PCR product was ligated into the pGEM-T Easy (Promega) vector to construct

pGEMB7sm. *whiB7* was excised from pGEMB7sm using NdeI/ BamHI and ligated into similarly digested pET19b to construct pETB7sm.

Construction of 10xHis:WhiB7 expression vector mutants

The QuickChange Lightning mutagenesis kit was used to mutate pETB7sm to change WhiB7's EPW sequence (aa 63-65) immediately upstream of the GVWGG turn. The sequence was mutated to the WhiB3 like EPY using the primers B7_EPY_F and B7_EPY_R to construct pETB7epy, and to the WhiB3 like VEY using the primers B7_VEY_F and B7_VEY_R to construct pETB7vey. The glutamate was also mutated to an aspartate using the primers B7_D_F and B7_D_R to construct pETB7d. The fourth cysteine (latter of the CXXC motif) was mutated to an alanine using the primers B7_48_F and B7_48_R to construct pETB748. Lastly, the CXXC motif cysteines were mutated to alanines using primers B7_4548_F and B7_4548_R to construct pETB74548.

Construction of WhiB7 and SigA co-expression vector

A C-terminal fragment of *M. smegmatis sigA* similar to that used to show WhiB3-SigA interaction (1), including regions 2.5, 3.1, 3.2, 4.1 and 4.2 (2), was amplified by PCR from pTRG170 (described below) using the primers CD_R42SM_F and CD_R42SM_R. This added a N-terminal strepII tag. The product was digested with NdeI/ KpnI, and cloned into similarly digested pColaDuet-1 to construct pSigA. 10xHis-tagged *whiB7* was extracted from pETB7sm using NcoI/ BamHI and cloned into similarly digested pSigA to construct the 10xHis-WhiB7 and strepII-SigA co-expression vector pSigAB7.

Construction of co-expression vector mutants

Mutant forms of 10xHis-WhiB7 were extracted by NcoI/ BamHI from pETB748, and pETB74548 and cloned into similarly digested pSigAB7 to construct pSigAB748 and pSigAB74548, respectively.

Construction of WhiB7 and region 4 of SigA co-expression vector

A similar alignment to that used by Vassylyev *et al.* (2) was used to identify the discreet regions of *M. smegmatis* SigA. The primers CD_R42short_F and CD_R42SM_R were used to

PCR amplify region 4 of SigA. The product was then digested with NdeI/ KpnI and cloned into similarly digested pSigAB7 to construct the 10xHis-WhiB7 and strepII-SigA(region 4) co-expression vector pR4B7.

Construction of Bait constructs

An AT-hookless *whiB7* was amplified by PCR from the *M. smegmatis* genome using the forward primer B7smF and the reverse primer B7smR_AT. The PCR product was digested with EcoRI and ligated into similarly digested pBT to construct pBTW7ΔC19. A 26 amino acid fragment of WhiB7, spanning the region downstream of the last cysteine and upstream of the AT-hook, was ordered from IDT (www.idtdna.com); B7midFrag_F and B7midFrag_R. The oligonucleotides were dissolved to 10 pmol/ μL and mixed at equal volumes. The mixture was heated to 95 ºC for 5 minutes and left to cool to room temperature. The resulting dimers were ligated into EcoRI/ XhoI digested pBT to construct pBTW7mid.

Construction of pBTW7ΔC19 bait mutants

The QuickChange Lightning mutagenesis kit was used to mutate pBTW7ΔC19 to change WhiB7's EPW sequence (aa 63-64) immediately upstream of the GVWGG turn. The sequence was mutated to the WhiB3 like EPY using the primers B7_EPY_F and B7_EPY_R to construct pBTW7epy, and to the WhiB3 like VEY using the primers B7_VEY_F and B7_VEY_R to construct pBTW7vey. The glutamate was also mutated to an aspartate using the primers B7_D_F and B7_D_R to construct pBTW7d. A glutamate further downstream (E71) was also mutated using the primers B7_71D_F and B7_71D_R to construct pBTW71d. The fourth cysteine (latter of the CXXC motif) was mutated to an alanine using the primers B7_48_F and B7_48_R to construct pBTW748. Lastly, the CXXC motif cysteines were mutated to alanines using primers B7_4548_F and B7_4548_R to construct pBTW74548.

Construction of pTRG170

sigA from *M. smegmatis* (MSMEG_2758) was amplified by PCR using the primers SigAsmF and SigAsmR. The PCR product was digested with EcoRI/ SpeI, and ligated into similarly digested pTRG to construct pSigASM. pSigASM was digested with EcoRI/ NcoI, and self-ligated. Sequencing confirmed that this resulted in a 901 bp deleted region spanning from

immediately downstream of the pTRG BamHI site to the ATG within the NcoI site in *sigA* leading to pTRG170. This expressed a C-terminal fragment of *M. smegmatis* SigA (aa 297-466) which is 100% similar (99.6% identical) to that of *M. tuberculosis* SigA (aa 359-528). Previous studies investigating WhiB3-SigA interaction used aa 369-528 as the SigA target (1).

Construction of a pTRG170 R515H mutant

The QuickChange Lightning mutagenesis kit was used to mutate pTRG170 with the primers mutR42_F and mutR42_R to construct pTRG170.515. This resulted in a MSMEG_2758 G1358A mutation leading to expression of SigA Arg453His. This corresponds to the *Mtb* SigA R515H mutation. The *Mtb* numbering will be used to simplify discussion of previous WhiB3-SigA results.

Construction of *M. smegmatis* Sig515

The R515H mutated region 4.2 of *sigA* was removed from pTRG170.515 using NcoI/ HindIII. It was combined with the XhoI/ NcoI *sigA* fragment from pSigASM and cloned into XhoI/ HindII digested pYUB854 to construct pSig515. The downstream region of *sigA* was then amplified by PCR from the *M. smegmatis* genome using the primers SigAflank_F and SigAflank_R. The PCR product was digested with XbaI/ KpnI and cloned into similarly digested pSig515 to construct pSig515KI. This reversed the orientation of the hygromycin resistance gene so that it matched the orientation of *sigA*. pSig515KI was then digested with XhoI/ KpnI and the digest was used to construct *M. smegmatis* Sig515 using mycobacterial recombineering (3) (Figure 5A). Three randomly picked recombinants were used for all determinations. *sigA* from the mutants was amplified using the primers SigAsmF and SigAsmR. The product was cloned, utilizing the 5' addition of A overhangs, into pGEM-T easy and sequenced to assure the R515H mutation. The presence of *whiB7* in these strains was confirmed by PCR using the primers 497_F and B7smR.

***In vitro* run-off templates**

Linear templates for *in vitro* transcription were prepared using PCR. The *whiB7* promoter (-103/+185) and AT rich region lacking promoter (-92/+185) were amplified from pMS497GFP and pMS483GFP, respectively, using the primers pMycB7 and B7+185 ('-'indicates the number

of bases upstream of the transcription start site, +1, and '+' indicates the number of bases downstream). Alternative templates with various upstream and downstream lengths were amplified from pMS689GFP using the primer combinations pB7GFP_6F/ B7+185, pB7GFP_7F/ B7+185, pB7GFP_6F/ R4D_R, and pB7GFP_7F/ R4D_R, yielding -85/ +185, -193/ +185, -85/ +225, and -193/ +225 templates. The *groEL2* promoter (-140/ +184) was amplified from pMV261 using the primers pMV261F and pMV261R. Templates were amplified by Econotaq (Lucigen). Reaction mixtures were according to manufacturer's instructions, supplemented with 1.5 mM $MgCl_2$ and 5% (v/v) DMSO. PCR condtions were: 95 °C for 5 min, 35 cycles (94 °C 20 s, 60 °C 20 s, 72 °C 20 s) and 72 °C for 5 min. PCR products were isolated with GenElute PCR cleanup kit (Sigma) according to manufacturer's instructions with the exception that the DNA was eluted with a solution of 10 mM Tris (pH 8.5) and 30 mM sodium acetate.

Purification of WhiB7

pETB7sm was transformed into *Escherichia coli* Rosetta2 (DE3) and plated on LB agar containing ampicillin (amp) and chloramphenicol (cm). A single transformant was inoculated into 30 mL of LB broth supplemented with amp, cm, and 0.2 % (w/v) dextrose (dex), and grown overnight. The culture was diluted 1/ 100 into two flasks containing 1 L LB supplemented with amp, cm and dex and grown for 2 hours at 37 ºC, 200 rpm to an OD 600 nm of about 0.2. The cultures were transferred to 16 ºC and incubated for 30 min at 200 rpm. Finally, isopropyl-beta-D-thiogalactopyranoside (IPTG) was added to a final concentration of 0.3 mM and the culture incubated for an additional 17 h at 16 ºC.

Cells were pelleted by centrifugation (3500 g) and the pellet was suspended in 20 mL lysis buffer (50 mM Na_2PO_4, 300 mM NaCl, 10 mM imidazole, 50 µg/mL phenylmethanesulphonylfluoride, 5 mM 2-mercaptoethanol, pH 8). The suspension was split equally (ca. 15 mL fractions) into two 50 mL conical tubes and sonicated on ice for 30 s twelve times with 1 min breaks using a CL4 sonicator (Mandel) at setting 4 (15 % power). The lysed cells were pelleted at 3500 g for 20 min and the supernatants combined (~30 mL) into an ultracentrifuge tube. The supernatant was centrifuged using an Optima L-90k (Beckman) with a Type 70 Ti rotor at 30, 000 rpm for 30 min at 4 ºC. The supernatant was then filtered through a 0.45 µm filter (Mandel 229749).

A 10 mL syringe was used as a column containing 1 mL of Ni-NTA resin (Qiagen) held at 4 ºC. The column was washed with 6 column volumes (CV) of wash buffer (50 mM Na_2PO_4 300 mM NaCl, 10 mM imidazole, pH 8) and the flow rate adjusted to 1 mL/ min. The filtered supernatant was then passed over the column resulting in the column turning brown. The column was then washed with six 10 CV loads of wash buffer containing an increasing amount of imidazole (50, 60, 70, 80, 90, and 100 mM). Finally elution buffer (50 mM Na_2PO_4, 300 mM NaCl, 250 mM imidazole, pH 8) was applied to the column. This resulted in a clear, brown eluate. Fractions which were visibly dark brown were pooled, DTT was added to a final concentration of 2 mM, and aliquots were immediately frozen in liquid nitrogen and stored at -80 ºC until use.

Supplementary Tables and Figures

Table S1. Oligonucleotides used in this study

FB7L_F	TAATAGGTACCAGACGGAGAATTCGTCATCG
FB7L_R	TAATACCATGGCAATGGACATGTGTTTTTCC
FB7R_F	TTAATACCATGGACTACAAGGACCACGATGGCGACTACAAGGACC ACGATATCGACTACAAGGACGATGACGACAAGATGACTGCTCCGA CCACG
FB7R_R	TAATTAAGCTTGATCTCCGAATGGATGGAAG
B7fun_F	TATTACCATGGTGCCGTGCCATGTCGG
B7fun_R	TAATCAAGCTTAGCCGGAATCCTTACGCGG
HSP60F	CAGGAGCATTGCCGTTCC
HSP60R_pst	TAATAACTGCAGTGCGAAGTGATTCCTCCG
FlagB7smF	TAATACTGCAGATGGACTACAAGGACCACGATGG
FlagB7smR	TAATCAAGCTTGGGGCGGTCGATCAGGC
FlagB7smR_AT	TAATCAAGCTTTCACGCGACAATGCTTCCGC
SRG-15	TGCCCATATGACTGCTCCGACCACGG
SRG-16	AAAGGATCCGATCAGGCGGCGGC
CD_R42SM_F	CATATGGCTAGCTGGAGCCACCCGCAGTTCGAAAAAGGCGCGATG GCCGACCAGGCCC
CD_R42SM_R	GGTACCTTACTAGTCCAGGTAGTCGCGCAGC
CD_R42short_F	CATATGGCTAGCTGGAGCCACCCGCAGTTCGAAAAAGGCGCGGTG GACGCCGTGTCGTTC
B7smF	TAATAGAATTCGACTGCTCCGACCACGGG
B7smR	TAATAGAATTCGGGGCGGTCGATCAGGC
B7smR_AT	TAATAGAATTCTCACGCGACAATGCTTCCGC
B7midFrag_F	AATTCGCTGACCGCGGCGCTCGAACGGCAGGAACCGTGGGGTGTC TGGGGTGGCGAGATCCTCGACCGCGGAAGCATTGTCGCGTGA
B7midFrag_R	TCGATCACGCGACAATGCTTCCGCGGTCGAGGATCTCGCCACCCCA GACACCCCACGGTTCCTGCCGTTCGAGCGCCGCGGTCAGCG
B7_EPY_F	GCGCTCGAACGGCAGGAACCGTACGGTGTCTGGGGTGGCGAG
B7_EPY_R	CTCGCCACCCCAGACACCGTACGGTTCCTGCCGTTCGAGCGC
B7_VEY_F	GCGCTCGAACGGCAGGTGGAGTACGGTGTCTGGGGTGGCGAG
B7_VEY_R	CTCGCCACCCCAGACACCGTACTCCACCTGCCGTTCGAGCGC
B7_D_F	GCGCTCGAACGGCAGGACCCGTGGGGTGTCTGGGGTGGCGAG
B7_D_R	CTCGCCACCCCAGACACCCCACGGGTCCTGCCGTTCGAGCGC
B7_71D_F	GGTGTCTGGGGTGGCGACATCCTCGACCGCGGAAGC
B7_71D_R	GCTTCCGCGGTCGAGGATGTCGCCACCCCAGACACC
B7_48_F	CCAAGGCCCTGTGCGCGGGGGCCCCGATCCGTGTGCAGTGCC
B7_48_R	GGCACTGCACACGGATCGGGGCCCCCGCGCACAGGGCCTTGG
B7_4548_F	CCTTGAGCGGGCCAAGGCCCTGGCCGCGGGGGCCCCGATCCGTGT GCAGTGCC
B7_4548_R	GGCACTGCACACGGATCGGGGCCCCCGCGGCCAGGGCCTTGGCCC GCTCAAGG
SigAsmF	TAATAGAATTCTGGCAGCGACAAAGGCAAG
SigAsmR	TAATCACTAGTCTAGTCCAGGTAGTCGCGCAGC

mutR42_F	GTCCAAGACCATGTCGAAGCTGCACCACCCGAGCCGTTCGCAGGT G
mutR42_R	CACCTGCGAACGGCTCGGGTGGTGCAGCTTCGACATGGTCTTGGAC
SigAflank_F	TAATGTCTAGACAGCCGTTCGCAGGTGCTGC
SigAflank_R	TAATAGGTACCGCTTGTCAGAGTTGCGTTCATGG
497_F	TACCATCGATGCAGGTAGAAAATAGGTTGTGCG
B7smR	TAATAGGATCCGGGGCGGTCGATCAGGC
pMycB7	TCTCGGCTCGATGATCC
B7+185	ACCAGCGGCGGCGTGAG
pB7GFP_6F	TAATACAACGTTCGTAAAGATCGTGCCAAAACC
pB7GFP_7F	TAATACAACGTTCCGACGACGCCGCACTC
R4D_R	TAATATCTGCAGGCGGCGCAATGGCAGC
pMV261F	TTACGGGTCTTGTTGTCGTT
pMV261R	ATTGCGAAGTGATTCCTCC
261seq_F	TTACGGGTCTTGTTGTCGTT
261seq_R	CCCGTTGAATATGGCTCATAAC
pBTseq_R	CCAGTTTGCTCAGGCTCTCC
pTRGseq_F	CATTCTGGCTGAACAACTGG
pTRGseq_R	ACGCTCAGTGGAACGAAAAC
pCDseq_F	CCCTTATGCGACTCCTGC

Table S2. Possible interactions between modeled *Mtb* WhiB7 and SigA. The letters in subscript indicate the labeling on the nitrogen and oxygen atoms in Molecular Operating Environment suite.

**Interaction type**	**WhiB7_MTB residue**	**SigA_MTB residue**
Hydrogen bond	R8.N_{H1}	S468.O_{G}
Ionic bond	R8.N_{H2}	E469.O_{E2}
Ionic bond	E61.O_{E2}	R515.N_{H2}
Ionic bond	E69.O_{E2}	R502.N_{H2}
Hydrophobic contact	F71.C_{E2}	I506.C_{D1}
Hydrogen bond	D72.O_{D2}	S468.O_{G}
Hydrogen bond	Q73.O_{E1}	K513.N_{Z}
Hydrogen bond	S75.O_{G}	E469.O_{E1}
Hydrogen bond	S75.N	E471.O_{E2}
Hydrogen bond	R81.N_{H1}	G497.O
Hydrogen bond	A90.O	R502.N_{H1}
Hydrogen bond	V91.O	Q505.N_{E2}
Hydrogen bond	A92.O	K509.N_{Z}

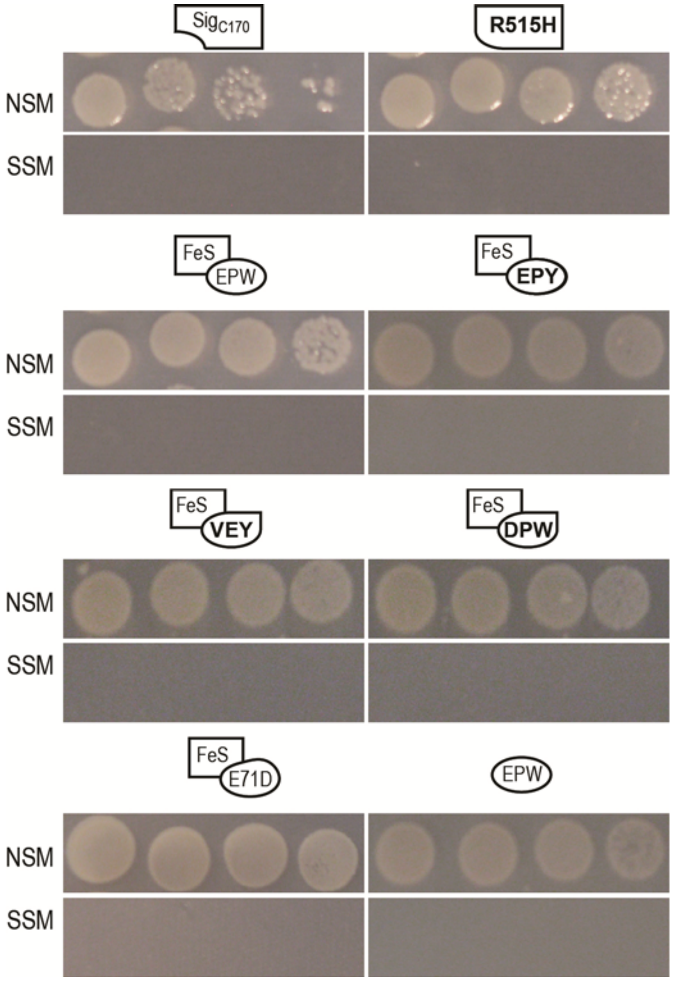

Figure S1. Neither bait nor target constructs can alone activate *HIS3* expression. BacterioMatch II two-hybrid assay results for bait and target constructs paired with an empty partner. The C-terminal fragment of SigA, ‘SigA$_{C170}$’, or its R515H mutant, ‘R515H’, are boxes with modified left sides. WhiB7 is represented in two parts: the cysteine iron binding box ‘FeS’ (aa 1-54) and the glycine rich tryptophan turn region oval ‘mid’ (aa 55-80). Mutations in the 'mid' region are bolded in the oval (EPY = W65Y; VEY = E63V P64E W65Y; DPW = E63D; E71D = E71D).

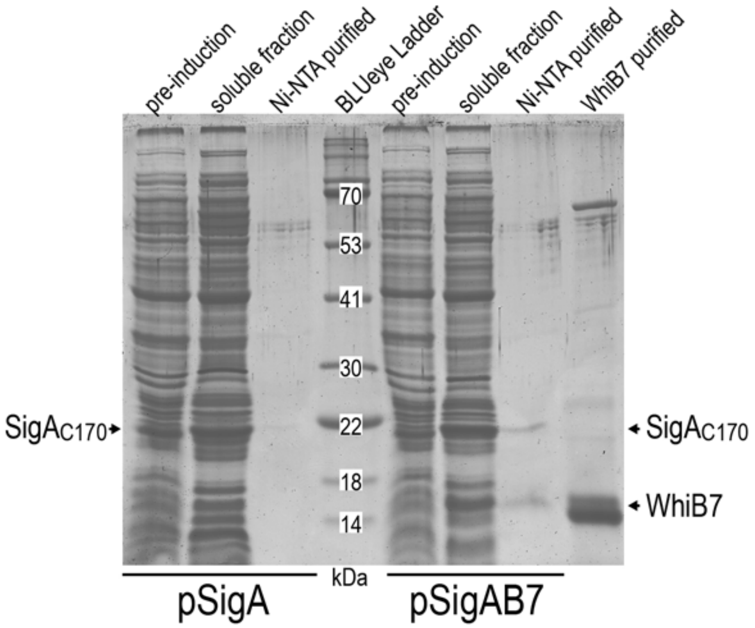

Figure S2. Batch co-purification of SigA by WhiB7 pull-down. A C-terminal fragment of SigA (SigA$_{C170}$) was expressed with (pSigAB7) and without (pSigA) WhiB7. Soluble proteins (soluble fraction) were passed through Ni-NTA resin and the retained protein eluted (Ni-NTA purified). The expected sizes are 20.9 kDa for SigA and 13.3 kDa for WhiB7. 10xHis-WhiB7 contains a very high proportion of positive amino acid residues (27/ 122) and was therefore expected to appear larger with SDS-PAGE separation. Pull-down of WhiB7 by Ni-NTA co-purified SigA (Ni-NTA purified pSigAB7). SigA expressed alone (pSigA) could not be purified by Ni-NTA. The WhiB7 purification used for *in vitro* run-off experiments is provided as a positive WhiB7 control (WhiB7 purification). The sizes of the ladder (BLUeye Ladder) are indicated (kDa).

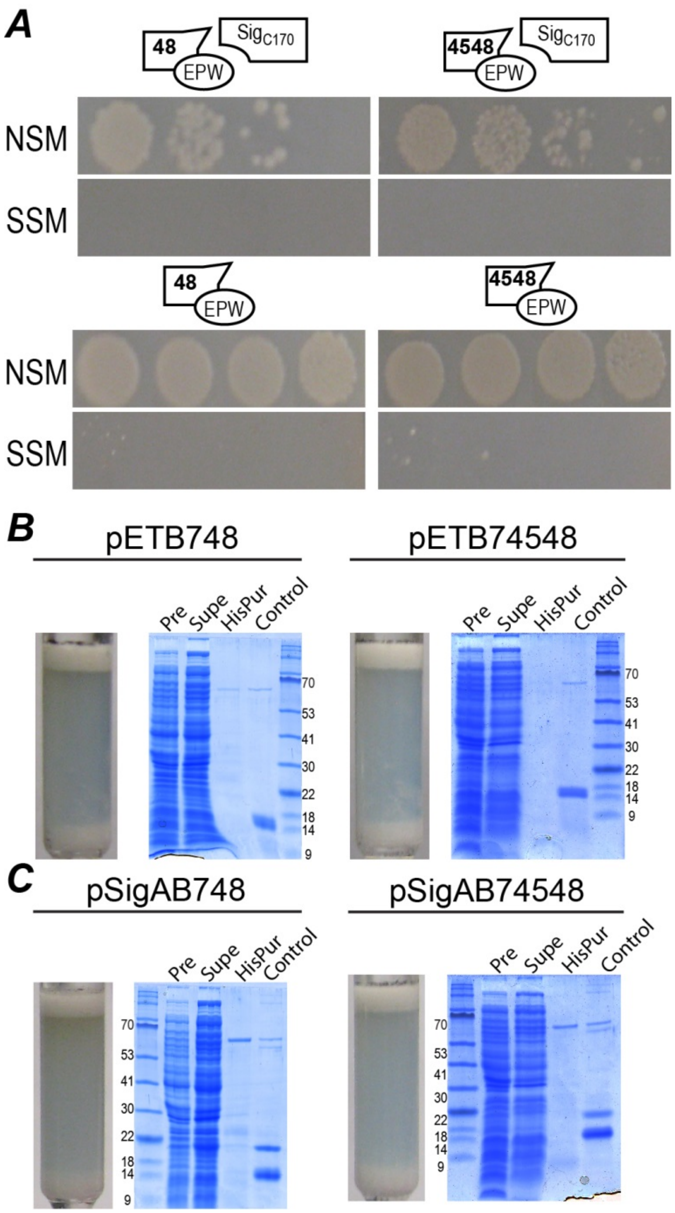

Figure S3. WhiB7s C48A and C45A C48A are unstable and interactions with SigA could not be monitored. (A) BacterioMatch two-hybrid results for cysteine mutants C48A (48) and C45A C48A (4548) in combination with SigA$_{C170}$ (top) or alone (bottom). No growth on selective screening medium (SSM) indicates no interaction. WhiB7 is represented in two parts: the cysteine iron binding box ‘FeS’ (aa 1-54) with the mutations indicated and the glycine rich tryptophan turn region oval ‘mid’ (aa 55-80). **(B)** Overexpression and purification of WhiB7 C48A (pETB748) and WhiB7 C45A C48A (pETB74548). The washed Ni-NTA resin was shown to remain light blue indicating that no FeS cluster containing protein was retained. A tricine SDS-PAGE gel shows the protein profiles of the purification steps including whole cell pre-

induced (Pre), soluble protein post-induction (Supe), Ni-NTA purified fraction (HisPur), and a purified WhiB7 control (Control). The approximate kDA of the ladder bands is indicated. **(C)** Co-expression and purification of WhiB7 C48A (pSigAB748) and WhiB7 C45A C48A (p CDR42B74548) with a C-terminal fragment of SigA containing region 4.2. The washed Ni-NTA resin was shown to remain light blue indicating that no FeS cluster containing protein was retained. A tricine SDS-PAGE gel shows the protein profiles of the purification steps including whole cell pre-induced (Pre), soluble protein post-induction (Supe), Ni-NTA purified fraction (HisPur), and a purified WhiB7 or WhiB7-SigA$_{C170}$ complex control (Control). The approximate kDA of the ladder bands is indicated.

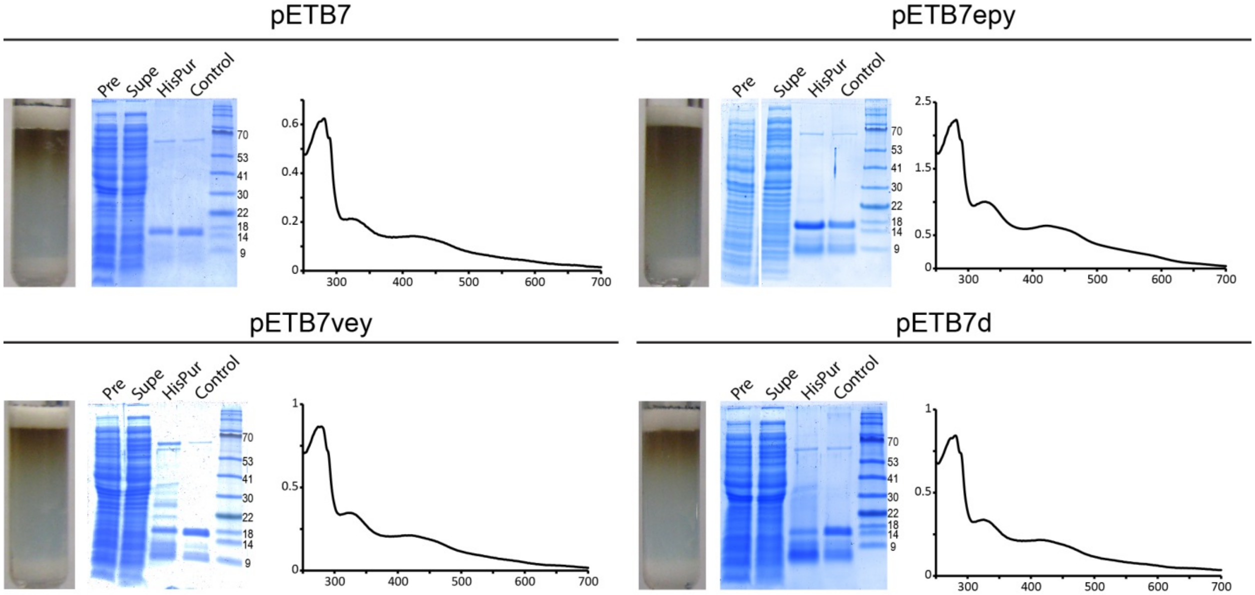

Figure S4. WhiB7 mutants express and bind an FeS cluster. Overexpression and purification of WhiB7 (pETB7), WhiB7 W65Y (pETB7epy), WhiB7 E63V P64E W65Y (pETB7vey), and WhiB7 E63D (pETB7d). The washed Ni-NTA resin was shown to contain a bound dark brown region which suggests a FeS cluster containing protein was retained. A tricine SDS-PAGE gel shows the protein profiles of the purification steps including whole cell pre-induced (Pre), soluble protein post-induction (Supe), Ni-NTA purified fraction (HisPur), and a purified WhiB7 control (Control). The approximate kDa of the ladder bands is indicated. Purification in all cases resulted in a purified protein matching WhiB7. WhiB7 E63V P64E W65Y may be prone to slight multimerization. Lastly, the absorption spectra of each construct contained a 350-400 nm shoulder and 400-450nm peak indicating the presence of a [2Fe-2S] FeS cluster.

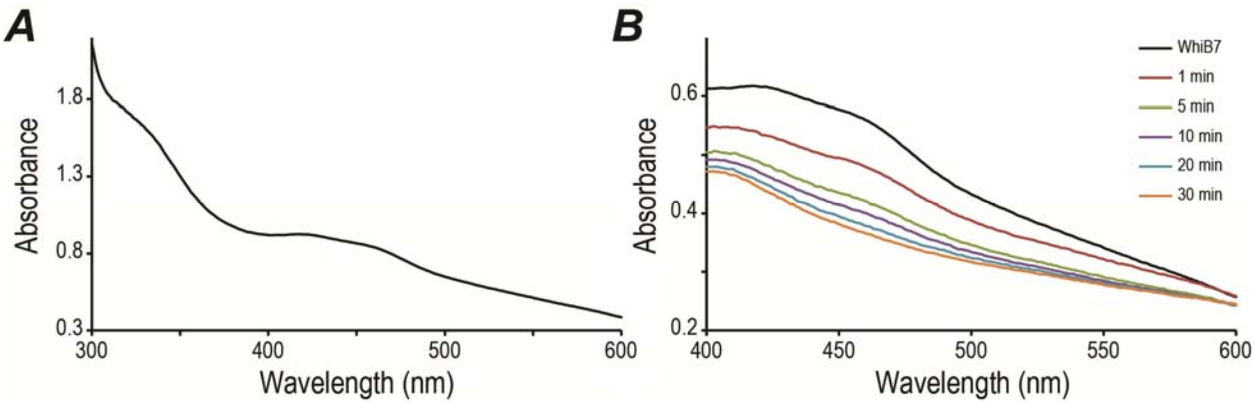

Figure S5. Absorbance spectra of purified 10xHis-WhiB7. (A) UV absorbance of purified 10xHis-WhiB7. A broad shoulder between 300-350 nm and a broad beak between 400-450 nm is indicative of [2Fe-2S] clusters. **(B)** UV absorbance time-course of diamide treated 10xHis-WhiB7. The purified protein was diluted 2/3 with a diamide stock solution to achieve a final concentration of 7 mM diamide. The corresponding curve from 'A' lowered by 2/3 is added (black line; 'WhiB7') for reference. Time points of the readings are indicated. The 450 nm shoulder decreases in a time dependant manner indicating a loss of the iron-sulphur cluster.

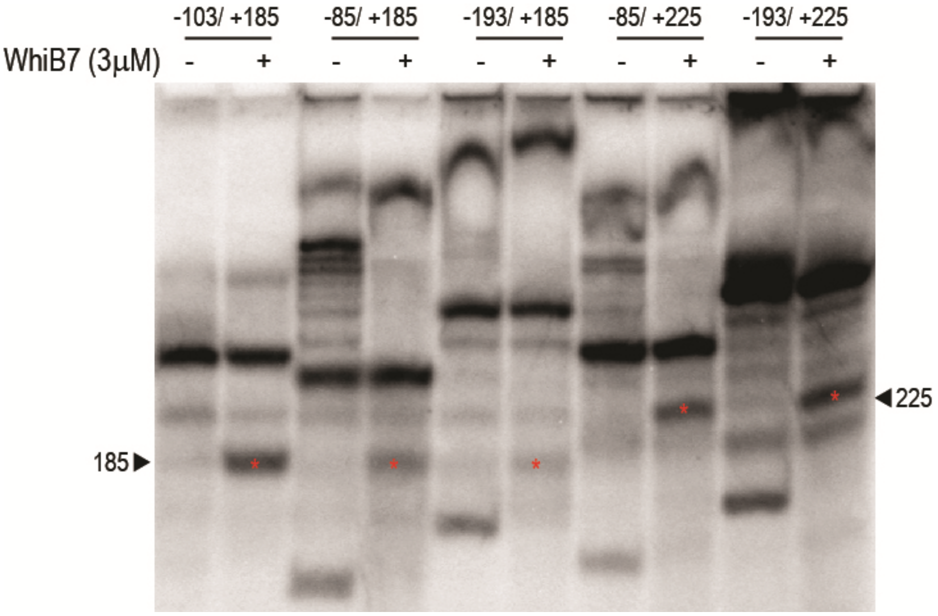

Figure S6. WhiB7 catalyzed run-off transcription from the predicted start site of the *whiB7* promoter. The size of the upstream (denoted by '-') and downstream (denoted by '+') lengths relative to the transcriptional start site (+1) of the templates are indicated. WhiB7, at a fixed concentration (3 μM), was added (+) or withheld (-). The products corresponding to the expected size of the whiB7 promoter transcript (185 or 225 bp) are highlighted by red stars. The other prominent bands represent non-specific transcripts.

Supplemental References

1. Steyn, A.J., Collins, D.M., Hondalus, M.K., Jacobs, W.R., Jr., Kawakami, R.P. and Bloom, B.R. (2002) Mycobacterium tuberculosis WhiB3 interacts with RpoV to affect host survival but is dispensable for in vivo growth. *Proc Natl Acad Sci U S A*, **99**, 3147-3152.
2. Vassylyev, D.G., Sekine, S., Laptenko, O., Lee, J., Vassylyeva, M.N., Borukhov, S. and Yokoyama, S. (2002) Crystal structure of a bacterial RNA polymerase holoenzyme at 2.6 A resolution. *Nature*, **417**, 712-719.
3. van Kessel, J.C. and Hatfull, G.F. (2008) Mycobacterial recombineering. *Methods Mol Biol*, **435**, 203-215.
